# Supplementary material for: A Critical View of the Application of the APEX Software (Aqueous Photochemistry of Environmentally-Occurring Xenobiotics) to Predict Photoreaction Kinetics in Surface Freshwaters
Source: Molecules. 2019 Dec 18;25(1):9. doi: 10.3390/molecules25010009 (PMC7017383; doi:10.3390/molecules25010009)
Supplement: Supplementary file 1 [file molecules-25-00009-s001.zip › 3.2.4_gcc-4.4.0.zip!/3.2.4_gcc-4.4.0/3.2.4_gcc-4.4.0/doc/octave/html/interpreter/Breakpoints.html]

Breakpoints - Untitled


Next: Debug Mode,
Previous: Leaving Debug Mode,
Up: Debugging


---

### 13.3 Breakpoints

Breakpoints can be set in any Octave function, using the `dbstop`
function.

— Loadable Function: rline = **dbstop** (func, line, ...)  
> Set a breakpoint in a function
>
> `func`: String representing the function name. When already in debug mode this should be left out and only the line should be given. `line`: Line number you would like the breakpoint to be set on. Multiple lines might be given as separate arguments or as a vector.
>
> The rline returned is the real line that the breakpoint was set at.
>
>
>
> **See also:** dbclear, dbstatus, dbstep.

Note that breakpoints cannot be set in built-in functions
(e.g., `sin`, etc.) or dynamically loaded function (i.e., oct-files). To
set a breakpoint immediately on entering a function, the breakpoint
should be set to line 1. The leading comment block will be ignored and
the breakpoint will be set to the first executable statement in the
function. For example

```
     dbstop ("asind", 1)
     => 27
```

Note that the return value of `27` means that the breakpoint was
effectively set to line 27. The status of breakpoints in a function can
be queried with the `dbstatus` function.

— Loadable Function: lst = **dbstatus** (func)  
> Return a vector containing the lines on which a function has
> breakpoints set.
>
> `func`: String representing the function name. When already in debug mode this should be left out.
>
>
>
>
> **See also:** dbclear, dbwhere.

Taking the above as an example, `dbstatus ("asind")` should return
27. The breakpoints can then be cleared with the `dbclear` function

— Loadable Function: **dbclear** (func, line, ...)  
> Delete a breakpoint in a function
>
> `func`: String representing the function name. When already in debug mode this should be left out and only the line should be given. `line`: Line number where you would like to remove the breakpoint. Multiple lines might be given as separate arguments or as a vector.
>
> No checking is done to make sure that the line you requested is really
> a breakpoint. If you get the wrong line nothing will happen.
>
>
>
> **See also:** dbstop, dbstatus, dbwhere.

These functions can be used to clear all the breakpoints in a function. For example,

```
     dbclear ("asind", dbstatus ("asind"));
```

A breakpoint can be set in a subfunction. For example if a file contains
the functions

```
     function y = func1 (x)
       y = func2 (x);
     endfunction
     function y = func2 (x)
       y = x + 1;
     endfunction
```

then a breakpoint can be set at the start of the subfunction directly
with

```
     dbstop (["func1", filemarker(), "func2"])
     => 5
```

Note that `filemarker` returns a character that marks the
subfunctions from the file containing them.

Another simple way of setting a breakpoint in an Octave script is the
use of the `keyboard` function.

— Built-in Function: **keyboard** ()  
— Built-in Function: **keyboard** (prompt)  
> This function is normally used for simple debugging. When the
> `keyboard` function is executed, Octave prints a prompt and waits
> for user input. The input strings are then evaluated and the results
> are printed. This makes it possible to examine the values of variables
> within a function, and to assign new values if necessary. To leave the
> prompt and return to normal execution type return or dbcont.
> The `keyboard` function does not return an exit status.
>
> If `keyboard` is invoked without arguments, a default prompt of
> debug>  is used.
>
>
>
> **See also:** dbcont, dbquit.

The `keyboard` function is typically placed in a script at the
point where the user desires that the execution is stopped. It
automatically sets the running script into the debug mode.
